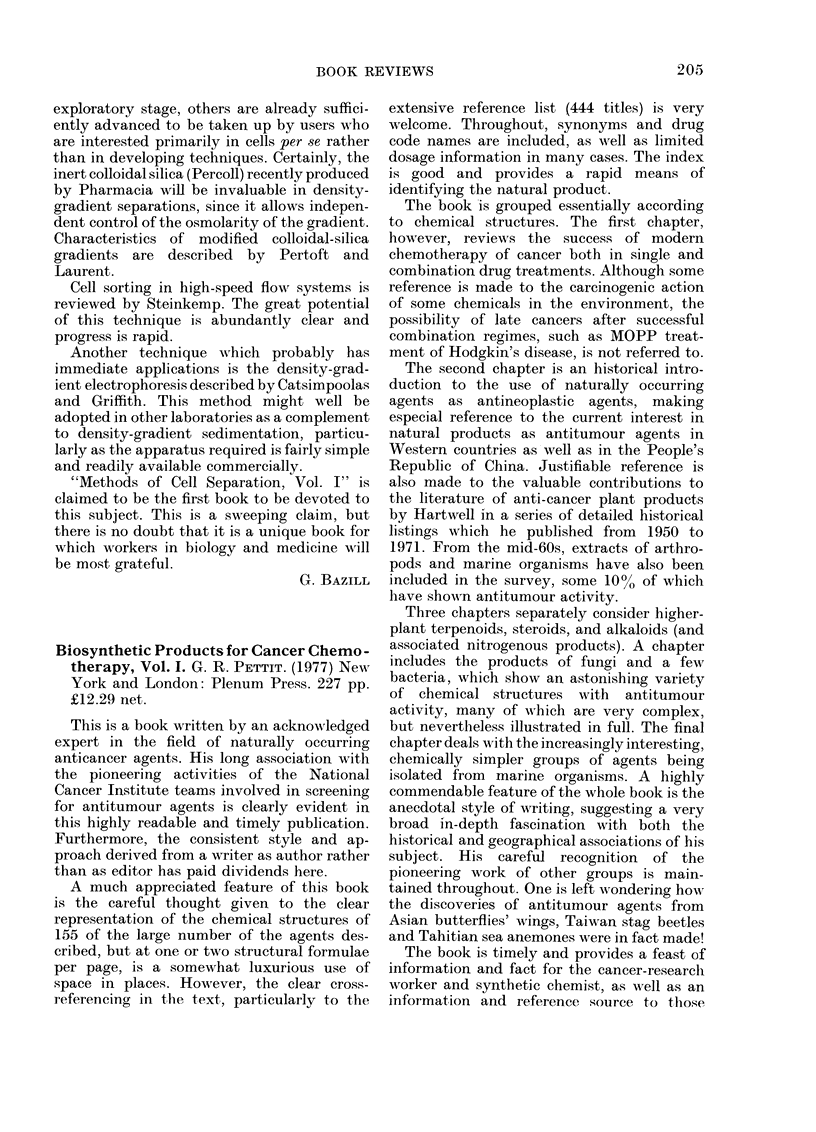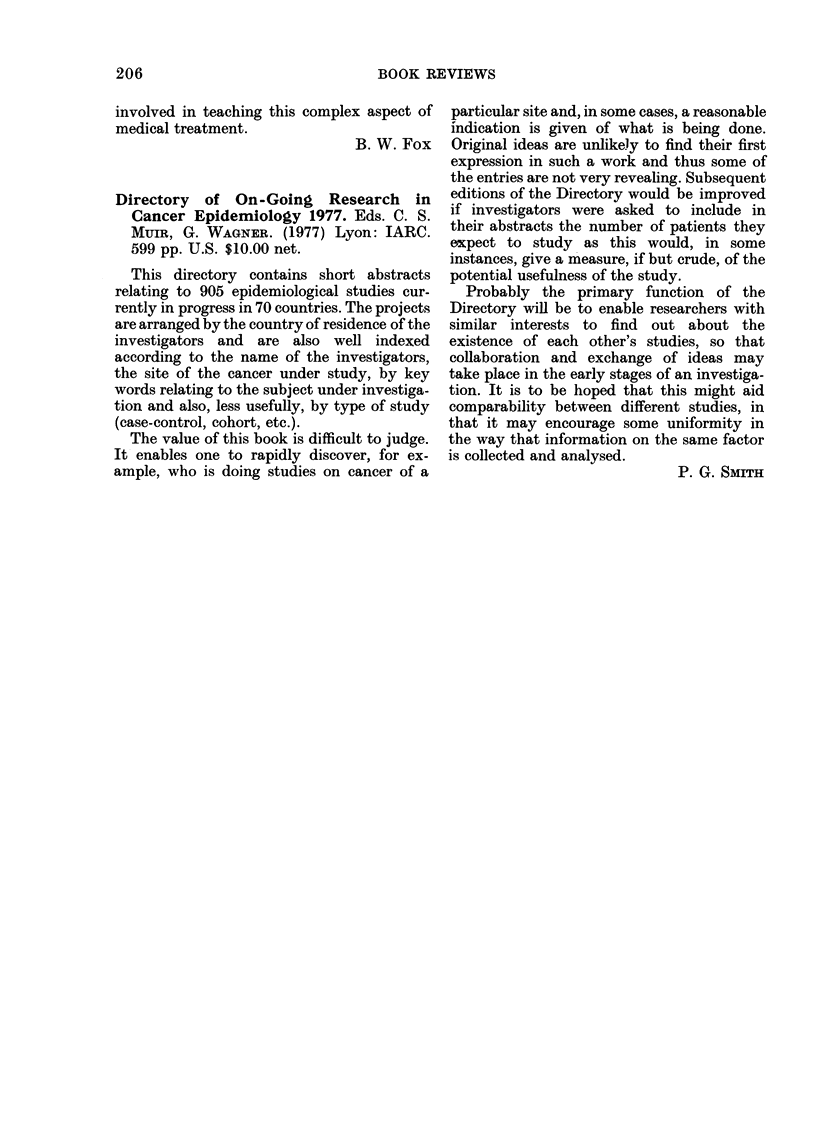# Biosynthetic Products for Cancer Chemotherapy, Vol. I

**Published:** 1978-07

**Authors:** B. W. Fox


**Biosynthetic Products for Cancer Chemotherapy, Vol. I.** G. R. PETTIT. (1977) New York and London: Plenum Press. 227 pp. £12.29 net.

This is a book written by an acknowledged expert in the field of naturally occurring anticancer agents. His long association with the pioneering activities of the National Cancer Institute teams involved in screening for antitumour agents is clearly evident in this highly readable and timely publication. Furthermore, the consistent style and approach derived from a writer as author rather than as editor has paid dividends here.

A much appreciated feature of this book is the careful thought given to the clear representation of the chemical structures of 155 of the large number of the agents described, but at one or two structural formulae per page, is a somewhat luxurious use of space in places. However, the clear cross-referencing in the text, particularly to the extensive reference list (444 titles) is very welcome. Throughout, synonyms and drug code names are included, as well as limited dosage information in many cases. The index is good and provides a rapid means of identifying the natural product.

The book is grouped essentially according to chemical structures. The first chapter, however, reviews the success of modern chemotherapy of cancer both in single and combination drug treatments. Although some reference is made to the carcinogenic action of some chemicals in the environment, the possibility of late cancers after successful combination regimes, such as MOPP treatment of Hodgkin's disease, is not referred to.

The second chapter is an historical introduction to the use of naturally occurring agents as antineoplastic agents, making especial reference to the current interest in natural products as antitumour agents in Western countries as well as in the People's Republic of China. Justifiable reference is also made to the valuable contributions to the literature of anti-cancer plant products by Hartwell in a series of detailed historical listings which he published from 1950 to 1971. From the mid-60s, extracts of arthropods and marine organisms have also been included in the survey, some 10% of which have shown antitumour activity.

Three chapters separately consider higher-plant terpenoids, steroids, and alkaloids (and associated nitrogenous products). A chapter includes the products of fungi and a few bacteria, which show an astonishing variety of chemical structures with antitumour activity, many of which are very complex, but nevertheless illustrated in full. The final chapter deals with the increasingly interesting, chemically simpler groups of agents being isolated from marine organisms. A highly commendable feature of the whole book is the anecdotal style of writing, suggesting a very broad in-depth fascination with both the historical and geographical associations of his subject. His careful recognition of the pioneering work of other groups is maintained throughout. One is left wondering how the discoveries of antitumour agents from Asian butterflies' wings, Taiwan stag beetles and Tahitian sea anemones were in fact made!

The book is timely and provides a feast of information and fact for the cancer-research worker and synthetic chemist, as well as an information and reference source to those involved in teaching this complex aspect of medical treatment.

B. W. Fox